# The Phase Change Heat of Water in the Pore Space of Rocks Based on DSC Studies

**DOI:** 10.3390/ma17164049

**Published:** 2024-08-14

**Authors:** Piotr Stępień, Edyta Spychał, Edyta Nartowska

**Affiliations:** 1 Faculty of Civil Engineering and Architecture, Kielce University of Technology, Al. Tysiąclecia Państwa Polskiego 7, 25-314 Kielce, Poland; espychal@tu.kielce.pl; 2 Faculty of Environmental Engineering, Geomatics and Renewable Energy, Kielce University of Technology, Al. Tysiąclecia Państwa Polskiego 7, 25-314 Kielce, Poland; enartowska@tu.kielce.pl

**Keywords:** rocks, DSC, thermoporometry, MIP

## Abstract

This research investigates the phase change behavior of water within the pore space of Devonian carbonate rock samples using Differential Scanning Calorimetry (DSC) across a temperature range of −80 to 0 °C. This study focuses on dolomite and limestone samples, all with porosities below 3%, an area not extensively covered in previous literature. Significant endothermic effects were observed at temperatures below −2 °C, challenging conventional understanding. The study reveals that the latent heat of phase change in these systems can exceed 334.2 J/g, the known value for bulk water, indicating unique thermodynamic properties of water in confined spaces. For the dolomite rock sample, observed endothermic heat effects below −2 °C were 23.5% and 26.7% of total phase change energy. The cumulative pore volume calculated using the thermoporometry method was found to be higher than expected from water occupancy alone, independent of assumptions about the thickness of the adsorbed unfreezable water layer or pore shape (spherical or cylindrical). This research provides novel insights into unfrozen water content calculations, significantly enhancing frost durability assessments and the geoengineering industry.

## 1. Introduction

Due to the abundance of deposits and the variety of applications, carbonate rocks are the subject of numerous scientific studies [1,2]. Depending on diagenesis, these rocks have diverse pore structures, open porosity and permeability. The amount of ice formed is crucial to understanding rock frost durability.

In porous materials, we can distinguish different types of water: those that undergo a phase change and those that do not. Under conditions of negative temperatures, part of the water remains in the liquid state; this water consists of unfrozen and non-freezable water content. The unfrozen water content corresponds to free water and weakly adsorbed water on a material surface. The amount of unfrozen water content decreases as the temperature decreases. Non-freezable water does not undergo a phase change to ice, even when temperatures drop below freezing. This fraction of water consist of water molecules strongly bound to the surface of the material and water in pores with a diameter under 2.4 nm [3,4]. Freezing of water is an exothermic process while melting is an endothermic process. The amount of heat released or supplied to the system during this change depends on the heat of phase change *L*(*T*). It is assumed that the latent heat of fusion of water is 334 kJ/kg at a temperature of 0 °C and normal atmospheric pressure (1 atm). The most common method for assessing the heat of phase change of water in a porous material at negative temperatures is differential scanning calorimetry (DSC) [5]. Knowledge of the unfrozen water content is used to assess the frost durability of mortars, concretes, rocks and ceramics, issues in geotechnical engineering, pavement engineering and food processing and research on the structure of porous materials using thermoporometry [6,7,8,9].

In materials where the freezing/melting phase change occurs at temperatures close to 0 °C, it seems sufficient to assume a constant value of the heat of phase change. An example of such a material may be building ceramic, with a dominant proportion of pores of micrometer size [10]. In other cases, the variation of the heat of phase change with temperature should be taken into account [11,12,13]. Empirical equations describing the dependence of the heat of phase change on temperature are still being sought. In addition to temperature, the value of the heat of phase change may depend on dissolved salts [14,15], clay content [16] and pressure [17]. In the literature, equations for the heat of phase change can be found, taking into account influence of the specific heat of water in the solid and liquid states on the entropy of the system [18]. This is how Equation (1) [18,19,20] is obtained:

(1) $$L\left(T\right)=334.1+2.119\cdot \Delta T-0.00783{\cdot \Delta T}^{2}$$

where: ΔT—difference between the temperature T and the temperature 273.2 K (K), *L*(*T*)—heat of phase change (J/g).

Equation (2) can be found in a simplified form [21,22]:

(2) $$L\left(T\right)=334.1+2.119\cdot \Delta T$$

In the literature, one can also find equations that derive a more significant decrease of the phase change heat value with increasing temperature depression than Formulas (1) and (2) [23]:

(3) $$L\left(T\right)=334+7.38\cdot \Delta T$$

Equation (3) was determined experimentally based on research on the energy associated with the ice melting in silica gels for various degrees of filling the pores with water. Analogous research was carried out by Litvan [24], examining the heat of phase change of water in silica gels for different thicknesses of water layers adsorbed on the pore surface, who found a drop in specific heat to 207 J/g for the case of freezing of two out of four layers of adsorbed water. In this research, with increasing layers of freezable water, the heat of phase change values approached those of free water. Similar studies were performed by Fung Kee Fung and Burke [25] for the melting of water in silica with pores of 6 nm, where they determined the value of the heat of phase change at 160.4 J/g, and for silica with pores of 15 nm at 241.2 J/g.

In addition to the previously given equations for the heat of phase change, the literature contains equations converted to the sum of freezing and non-freezable adsorbed water *L*′(*T*) according to Equation (4) [24,26]

(4) $$L’\left(T\right)=L\left(T\right)\frac{V_{freezable}}{V_{nonfreezable}+V_{freezable}}$$

where *V_freezable_*—volume occupied by freezing water (cm^3^) and *V_nonfreezable_*—volume occupied by non-freezable water strongly adsorbed to the pore surface (cm^3^);

Brun et al. [26] thus obtained Equation (5) for solidification

(5) $$L\left(T\right)=334+7.42\cdot \Delta T+0.0556{\cdot \Delta T}^{2}$$

And Equation (6) for melting

(6) $$L\left(T\right)=334+11.39\cdot \Delta T+0.0155\cdot \Delta T^{2}$$

Kozłowski [27] obtained a similar equation when examining the melting of ice in clay–water systems by comparing the volumes obtained through thermoporometry with the volumes from the method of Barett, Joyner and Halend (BJH) using nitrogen adsorption method at 77 K.

(7) $$L\left(T\right)=334+9.524\cdot \Delta T+0.068{\cdot \Delta T}^{2}$$

As Kozłowski shows [28], when examining the phase change of water in bentonites, adopting various assumptions regarding the phase change heat, different thicknesses of the non-freezable water layer were obtained, from 0.416 to 0.536 and from 0.296 to 0.363 nm, depending on the soil tested. The greatest thickness of the non-freezable adsorbed water layer was obtained by assuming a phase change heat value of 333.62 J/g.

Another factor influencing the value of phase change heat is the pressure generated by interfacial curvature on entropy values [17,26]. Based on calculations, it has been determined that for a cylindrical pore the heat of phase change of melting should be:

(8) $$L\left(T\right)=333.8+1.797\cdot \Delta T$$

For a spherical pore, Equation (7) for freezing and (9) for melting should be used.

(9) $$L\left(T\right)=332.4$$

Knowing the heat of phase change and the thickness of non-freezable adsorbed water, it is possible to determine the pore size distribution using the thermoporometric method. This method uses the Gibbs–Thomson equation, which allows the assignment of the phase change heat reduction temperature to the pore radii (10).

(10) $$\frac{\Delta T}{T_{0}}=\frac{2\gamma_{cl}cos\theta }{v_{m}L\left(T\right)r_{cl}}$$

where *γ_cl_* —surface tension at the solid-liquid interface (N/m), *L*(*T*)—heat of phase change (J/kg), *θ*—contact angle, *r_cl_*—radius of liquid-solid interface curvature (m) and *ν_m_*—liquid density (kg/m^3^).

The thermoporometric method requires knowledge of the heat of phase change, the non-freezable adsorbed water’s thickness, the filling substance’s density and assumptions about the shape of the pores. By comparing the results obtained from the thermoporometric method with other methods of examining the pore space, it is possible to calculate the amount of unfrozen water in the system and to determine the relationship between the pore radius and the freezing temperature. Ishikiriama et al. [19,29,30], using Equation (3) for the heat of phase change depending on the adopted pore shape (cylindrical or spherical), obtained different values of the thickness of non-freezable adsorbed water ranging from 0.3 nm to 1.77 nm. Sun et al. [17] and Brun et al. [26] assumed the thickness of this layer to be 0.8 nm.

Differential scanning calorimetry tests available in the literature are used primarily to determine the amount of unfrozen water content in pastes, mortars, concretes, soils and polymers [9,14,18,31,32,33,34,35,36,37,38]. The phase change of water itself at temperatures below −20 °C is also insufficiently recognized. Only a few publications use the DSC method to examine rock materials. The possibility of using thermoporometry to study the pore space of rocks with porosity below 3% is mainly unknown. Examples of such rocks are limestones and dolomites from the Świętokrzyskie Voivodeship from the Devonian period. They are characterized by one of the lowest porosities compared to limestones and dolomites from other regions of Poland [39]. Understanding their properties is crucial for assessing their strength and frost resistance, which is important for their application in construction and other engineering fields.

For this reason, in this research, the authors selected carbonate rocks with a porosity of less than 3%, for which they investigated water phase change at temperatures from −80 to 0 °C using differential scanning calorimetry. In addition, pore size distribution was tested using the Mercury Intrusion Porosimetry (MIP) method, and the basic physical properties of the materials were determined. These tests aimed to verify assumptions regarding the assumed values of phase change heat, the thickness of the non-freezable adsorbed water layer and the results obtained by the thermoporometry (TMP) method.

## 2. Materials and Methods

### 2.1. Materials

Three Devonian carbonate rocks from the Świętokrzyskie Region were used for investigation. One rock block of a volume of approximately 14 dm^3^ was collected from a quarry for each rock. The rocks were marked as follows: dolomite (DO1), limestone (CA1) and limestone (CA2). Dolomite DO1 and limestone CA2 come from the northern Paleozoic core of the Świętokrzyskie Mountains (Poland), while CA1 come from the southern and southwestern Paleozoic core of the Świętokrzyskie Mountains (Poland).

### 2.2. Methods

#### 2.2.1. Methods for Determining Physical Characteristics

A specific density test was performed using a Le Chatelier flask. Three markings were made on fragments from different parts of the rock. The rock was crushed in a mortar and the particles passed through a 0.08 mm sieve. The samples were dried to constant weight at 105 °C. The flask was filled with ethyl alcohol to the 0 level. A quantity of 21 to 22 cm^3^ of crushed rock was poured into the flask. The mass of the rock poured into the flask was determined on a scale with an accuracy of 0.01 g. The final result is the arithmetic mean obtained on three measurements.

The research on bulk density, specific density and porosity was carried out using the hydrostatic method. The hydrostatic method included: (1) drying the sample to a constant mass at a temperature of 105 °C, (2) weighing the sample to obtain a dry mass, (3) placing the sample in a sealed container and creating a pressure of 50 hPa, (4) soaking the sample under vacuum with distilled water at a temperature of 80 °C, (5) storing the sample in water for 5 days, (6) after complete saturation with water, removing the sample from the water, removing excess water with a damp cotton cloth and weighing again to obtain the mass of the water-saturated sample, (7) immersing the saturated sample in distilled water and weighing it to obtain the mass of the sample in water and (8) calculation of scores according to the formulas:

(11) $$V_{sample}=\frac{m_{n}-m_{hydr}}{\rho_{w}}$$

where *V_sample_*—volume of the sample (m^3^), *m_n_*—mass of the sample saturated with water (kg), *m_hydr_*—mass of the sample in water (kg) and *ρ_w_*—density of water (kg/m^3^).

(12) $$\rho_{0}=\frac{m_{s}}{V_{sample}}$$

where *ρ*_0_—bulk density (kg/m^3^) and *m_s_*—mass of the dry sample (kg).

(13) $$P=\frac{m_{n}-m_{s}}{V_{sample}}$$

where *P*—porosity (%).

#### 2.2.2. Differential Scanning Calorimetry (DSC)

The tests were carried out on cylindrical samples 13.5 mm in diameter and 65–70 mm in height. Samples were core-drilled with a diamond drill. Two specimens were prepared of each rock. Before testing, the samples were dried at 105 °C and soaked using the vacuum method with degassed distilled water. The soaked samples were stored in water for 7 days. Thermal signals during cooling and heating were recorded using a BT 2.15 CS differential scanning calorimeter (Setaram Instrumentation, Caluire, France). The scanning program included cooling the sample from +20 °C to −80 °C, then, after half an hour of stabilization at −80 °C, the sample was heated again to +20 °C. In the pore space, thermal exothermic signals are observed down to −55 °C. The scanning rate was 0.09 °C/min.

To eliminate the influence of the thermal inertia of the measurement system on the recorded heat fluxes, the procedure described in paper [40] was used. The methodology is based on successive approximations based on a set of apparatus functions. In this way, energy distributions assigned to appropriate temperatures were obtained, and, based on them, the total amount of melting ice was calculated by Equation (14):

(14) $$m_{ice-total}=\sum \frac{E_{i}}{L\left(T_{i}\right)}$$

where *E_i_* is the amount of energy of phase change at temperature *T_i_* (J) and *L*(*T_i_*)—value of heat of phase change at temperature *T_i_* (J/g).

Calculations of the mass of melting ice were made for two variants. Option A—calculation of the mass of melting ice using a constant value of 334.2 J/g for the phase change heat. In variant B, Equation (8) was adopted.

Additionally, as part of calorimetric tests, an experiment was carried out to determine the possible impact of dissolved substances originating from the sample skeleton on the heat of phase change of water. For this purpose, a fragment of rock DO1 weighing 25 g was crushed into particles no larger than 4 mm in size. The crushed rock was then mixed with 25 g of distilled water. This mixture was left aside for 10 days. Before the test, 0.6893 g of water was collected from the sediment and placed in a polyethylene test tube. The scanning program included cooling the sample from +20 °C to −20 °C, then after half an hour of stabilization at −20 °C, the sample was heated again to +20 °C. The scanning rate was 0.09 °C/min. Similarly, the phase change heat of 0.6893 g of distilled water was tested. 329.9 J/g for distilled water and 333.3 J/g for the solution were obtained. The performed experiment excludes the influence of dissolved substances in water on increasing the heat of phase change.

In order to determine sample volumetric absorption in DSC tests, the sample volumes V_sample_ were calculated using apparent density *ρ*_0_ based on the MIP test according to Equation (15) and water volume *V_l_* (16). The volumetric absorption *n_v_* was calculated based on Equation (17).

(15) $$V_{sample}=\frac{m_{s}}{\rho_{0}}$$

(16) $$V_{l}=\frac{m_{w}}{0.999\;g/{cm}^{3}}$$

(17) $$n_{v}=\frac{V_{l}}{V_{s}}$$

where *V_sample_*—volume of the sample (m^3^), *ρ*_0_—apparent density (kg/m^3^), *V_l_*—water volume in sample (m^3^) and *n_ν_*—volumetric adsorption (%).

#### 2.2.3. Thermoporometry

Pore size distribution was calculated based on melting energy distribution obtained from DSC measurements. The volume occupied by the melting of ice in specific temperature Δ*V_ice_*(*T_i_*) was calculated according to Equation (18).

(18) $$\Delta V_{ice}(T_{i})=\frac{E_{i}}{L\left(T_{i}\right)\rho_{ice}}$$

where *E_i_* is the amount of energy of phase change at temperature *T_i_* (J), *L*(*T_i_*)—value of heat of phase change at temperature *T_i_* (J/g) and *ρ_ice_*—density of ice (kg/m^3^).

The pore volume was calculated according Equation (19).

(19) $${\Delta V}_{pore}={\Delta V}_{ice}(T_{i}){\left(\frac{r_{p}+\delta }{r_{p}}\right)}^{n}$$

where *r_p_*—pore radius (m), n—shape factor, 2 for cylindrical pores and 3 for spherical and *δ*—thickness of an adsorbed water layer (m).

Equation for relation for pore radius and temperature of phase change obtained by Brun et al. [26] were used

(20) $$r_{p}=-\frac{32.33}{\Delta T}+0.68\;\;\;\;(\mathrm{nm})$$

#### 2.2.4. Mercury Intrusion Porosimetry (MIP)

Pore size distribution, porosity, density and bulk density were tested in an AutoPore IV model 9500 mercury porosimeter (Micromeritics, Norcross, GA, USA). Before testing, the sample was dried to constant weight at 105 °C. The sample was then cooled to 20 °C in a desiccator. Before testing, the sample was weighed and placed in the low-pressure port. The test included: (1) creating a vacuum in the penetrometer with the sample to the level of 2.6 Pa (20 μm Hg); (2) pouring mercury into the sample; (3) gradually increasing the pressure to 414 MPa while the apparatus measures the amount of mercury pressed and (4) gradually reducing the pressure to ambient values while the apparatus measures the amount of mercury exiting. For the calculations, a contact angle for mercury *θ* equal to 130° and a surface tension *γ_i_* equal to 0.485 N/m were assumed.

#### 2.2.5. Scanning Electron Microscopy (SEM)

The measurements were caried out using QUANTA FEG 250 scanning electron microscope equipped with an EDS EDAX detector (FEI Company, Eindhoven, The Netherlands). Rock fractures were studied at a magnification of 800. The results are given in Appendix A.

#### 2.2.6. X-Ray Diffraction

A PANalytical Empyrean diffractometer (PANanalitycal, Almelo, The Netherlands) with a Cu anode was used. The scan covered an angular range of 5 to 70° 2θ, using a step size of 3.3° min^−1^. The ICDD PDF-2 database was used as a reference database for the diffractogram analysis. The results are given in the Appendix B.

#### 2.2.7. Transmitted Light Optical Microscopy

Microscope analyses were conducted to determine grain size distribution. Analyses were carried using a semi-automatic microscope Optiphot-2 (Nikon Instruments Inc., Melville, NY, USA) with a digital camera in transmitted light. Samples were cut into slices on a diamond saw. The slices were ground, polished and glued to a measurement glass. In each sample 1000 mineral grains were counted.

## 3. Results and Discussion

### 3.1. Characteristics of the Rock Samples

Table 1 contains a macroscopic and mineralogical sample description based on SEM, X-ray diffraction and microscope analyses. In Appendix A, the micrograph of the studied rocks is shown.

### 3.2. Physical Properties

Table 2 summarizes the test results for specific density, bulk density and porosity. The physical property tests are consistent with the parameters that characterize Devonian carbonate rocks from the Świętokrzyskie Voivodeship [41].

### 3.3. Thermal Effects

**Figure 1:**
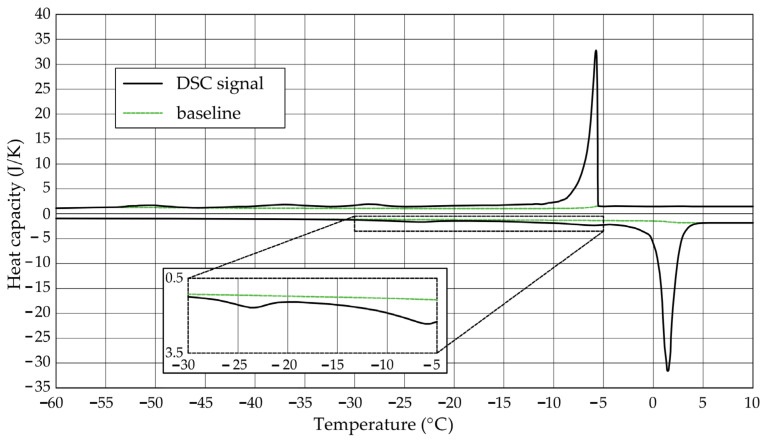
Heat capacity in the DSC test—sample DO1.1.

**Figure 2:**
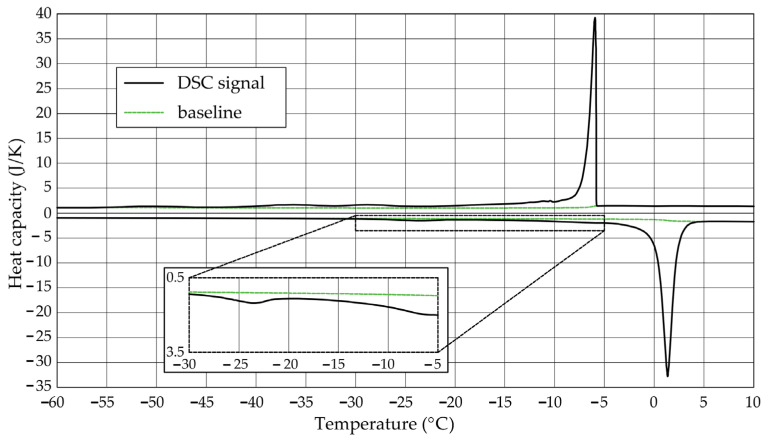
Heat capacity in the DSC test—sample DO1.2.

**Figure 3:**
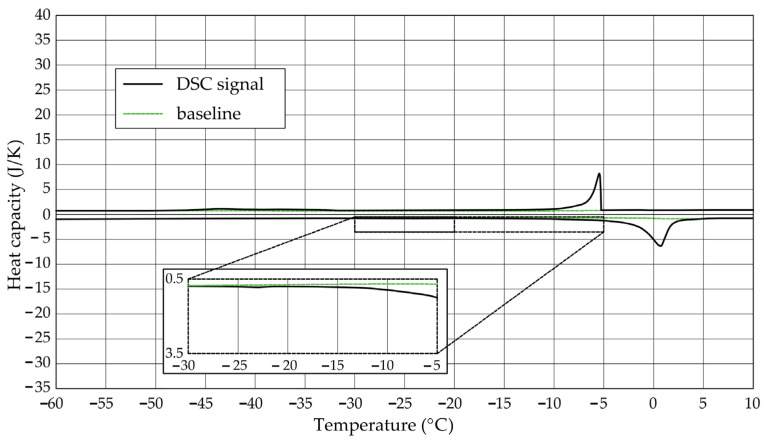
Heat capacity in the DSC test—sample CA1.1.

**Figure 4:**
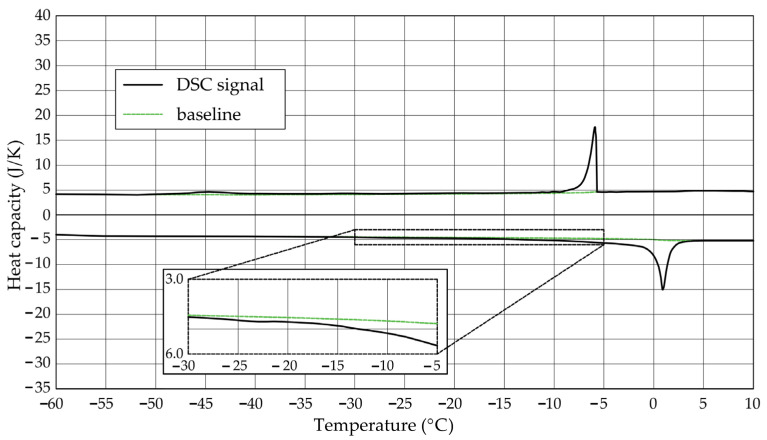
Heat capacity in the DSC test—sample CA1.2.

**Figure 5:**
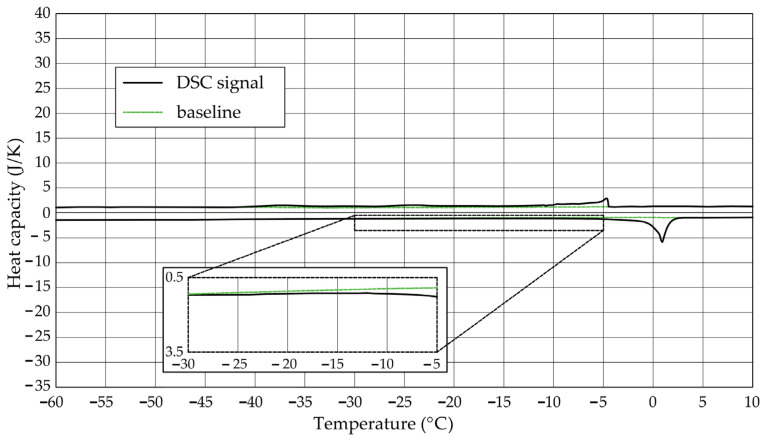
Heat capacity in the DSC test—sample CA2.1.

**Figure 6:**
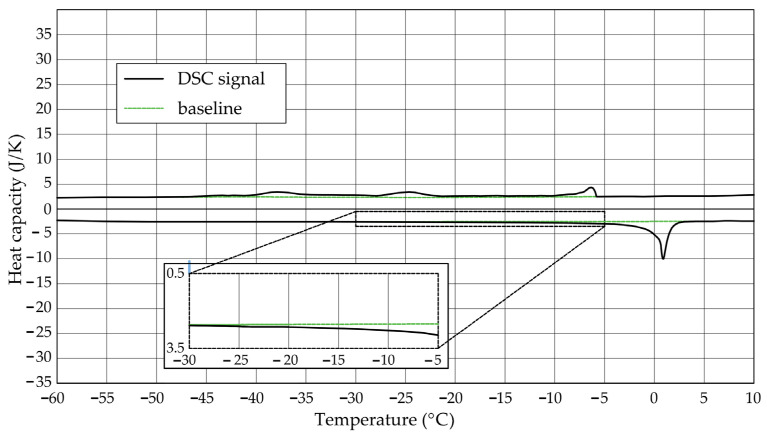
Heat capacity in the DSC test—sample CA2.2.

The Figure 1, Figure 2, Figure 3, Figure 4, Figure 5 and Figure 6 show the recorded heat capacities. Endothermic signals (related to the ice melting) were used to determine the amount of water freezing. In the case of the rocks examined (Table 3), three characteristic melting stages are observed. The first endothermic peak was recorded in the temperature range from −30 to −20 °C. The second stage involves the gradual melting of ice at temperatures above −20 °C. The last stage is related to the melting of water in the largest pores. The total amount of phase change energy and the amount of energy in individual temperature ranges are given in Table 4.

The calculated amount of ice determined based on the recorded signals is included in Table 5. For sample DO1, regardless of the adopted values of the phase change heat (variant A and B), the total ice mass (*m_ice-total_*) was always greater than the mass of water. In the case of rock CA1, this ratio is equal to or close to 1. In addition to freezable water, rocks will also contain non-freezable water, so values equal to 1 or greater than 1 may indicate higher values of energy needed for the phase change than those found in the literature or changes other than liquid–solid.

The calculated ice-to-water mass ratio above one may indicate specific interactions between water and the pore surface. Higher values of energy needed for phase change may result from:

- The influence of narrow pores on increasing the capillary pressure in the liquid. Higher capillary pressure can change the phase change temperature of the fluid (e.g., freezing point), which in turn affects the heat of fusion.
- In narrow pores, fluid molecules are strongly adsorbed on the pore walls, which changes their thermodynamic properties. These surface interactions can lead to a change in the phase change heat compared to free water [42].
- In very narrow pores, fluids can adopt molecular structures different from those in the free state. This change in structure can affect the energy required for a phase transition, often increasing the heat of the phase transition.

**Figure 7:**
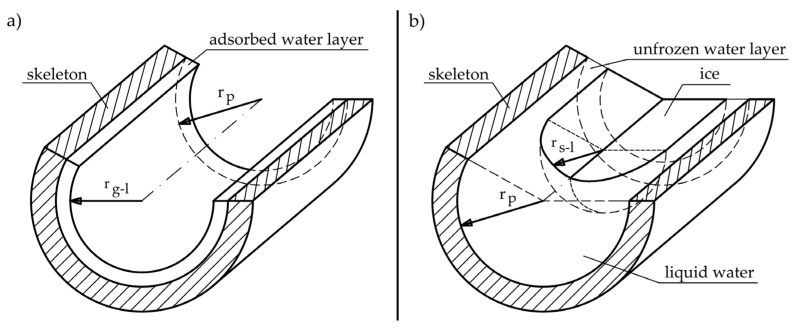
Phase interface in pores (**a**) liquid–gas interface for partly saturated cylindrical pore, where *r_p_*—pore radius and *r_gl_*—radius of the gas–liquid interface; (**b**) liquid–solid interface in fully saturated cylindrical pore, where *r_sl_*—radius of the solid–liquid interface.

The question arises as to why the equations in world literature for heat of phase change result in significantly lower values in subzero temperatures than those for free water. When we are dealing with a cylindrical pore, partially filled with water, which is located only on the surface of the walls (see Figure 7a), the change will be determined by the water–gas interfacial curvature which depends on pore shape in accordance with Equation (21).

(21) $$ln\left(\frac{T-T_{0}}{T_{0}}\right)=-\frac{\gamma_{cg}}{\rho_{l}L(T)}\frac{dA}{dV}=-\frac{1}{\rho_{l}L(T)}\left(\frac{1}{r_{1}}+\frac{1}{r_{2}}\right)$$

where *T*_0_—temperature 273.2 K, *γ_cg_*—surface tension on the gas and solid interface (N/m), *ρ_l_*—density of liquid (kg/m^3^) and *r*_1_*, r*_2_—radii of interface curvature (m).

In cylindrical pores, partly filled with water due to the shape of the pore (Figure 7a), there is curvature in only one direction and the term 1/*r*_2_ tends to zero.

When water freezes in a completely filled pore, the phase change will be determined by the liquid–solid surface (Figure 7b) in accordance with Equation (22) [43].

(22) $$ln\left(\frac{T-T_{0}}{T_{0}}\right)=-\frac{\gamma_{cl}}{\rho_{l}L(T)}\frac{dA}{dV}=-\frac{1}{\rho_{l}L(T)}\left(\frac{1}{r_{1}}+\frac{1}{r_{2}}\right)$$

where, *γ_cl_*—surface tension on the liquid–solid interface (N/m).

Therefore, it should be assumed that the phase change heat values given by [23,24,25] are appropriate in the case of testing the phase change of water when water occupies a couple of monolayers in the pore surface. There may be two reasons for obtaining significantly lower values for the heat of phase change in the case of Figure 7a than those obtained in the case of Figure 7b:

(1) Adsorbed water forms a partially ordered structure due to interactions between water molecules and the pore surface. These interactions reduce the energy needed to change the phase of water, resulting in a lower heat of phase change.
(2) The thickness of the non-freezable adsorbed water layer is more significant when the pores are filled with water than when there are only a few layers of water on the pore surface. Non-freezing water is closely bound to the pore surface, and the amount of it depends on the degree of pore filling. When the pores are completely filled with water, more water interacts with the pore surface, which increases the thickness of the non-freezing water layer. As a result, the heat released during the phase change is converted into a less significant mass of freezable water.

The results for rock CA2 show that the presence of a ratio of the calculated mass of ice to the mass of water (Table 5) greater than 1 is not a characteristic feature of Devonian carbonate rocks with porosity below 3%. This rock is also characterized by a significant share of exothermic thermal effects in temperatures below −10 °C (related to water freezing), similarly to rock DO1. Therefore, the calculated *m_ice-total_*/*m_w_* ratio for rock DO1 cannot be attributed to the influence of pore interconnections on the heat of phase change values.

### 3.4. Thermoporometry—Pore Size Distribution

**Figure 8:**
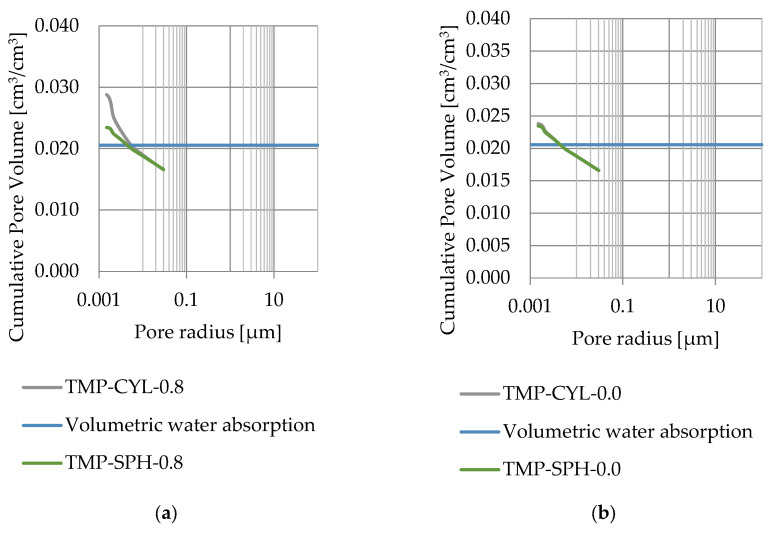
Cumulative pore volume—sample DO1.1: (**a**) 0.8 nm thickness for the nonfreezable layer, (**b**) 0.0 nm thickness for the nonfreezable layer.

**Figure 9:**
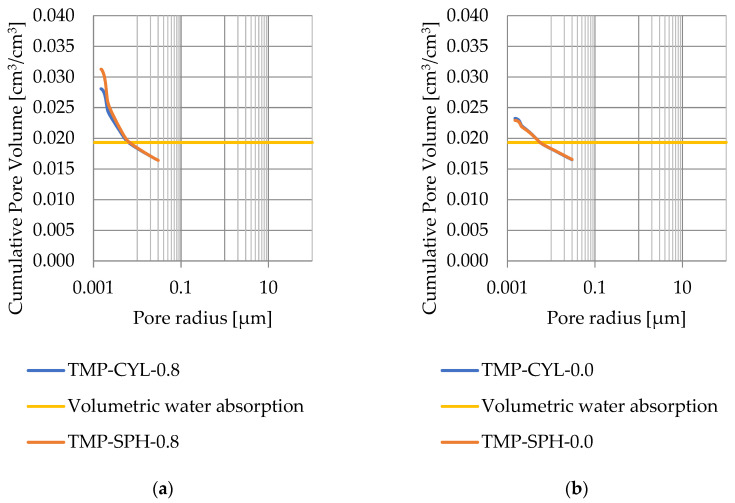
Cumulative pore volume—sample DO1.2: (**a**) 0.8 nm thickness for the nonfreezable layer, (**b**) 0.0 nm thickness for the nonfreezable layer.

**Figure 10:**
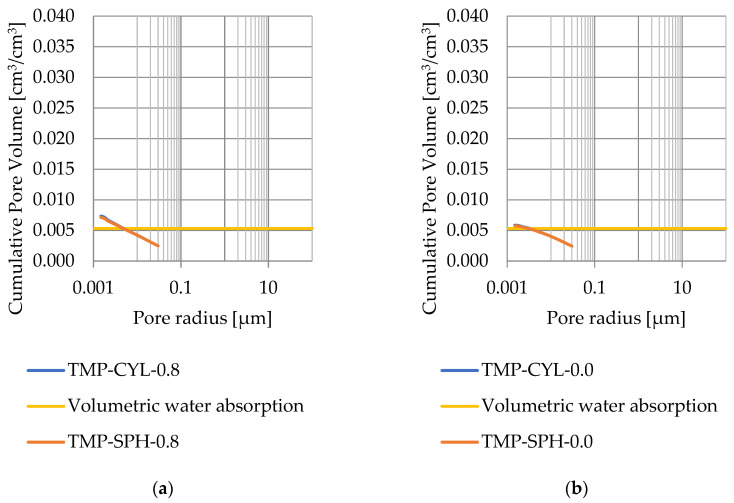
Cumulative pore volume—sample CA1.1: (**a**) 0.8 nm thickness for the nonfreezable layer, (**b**) 0.0 nm thickness for the nonfreezable layer.

**Figure 11:**
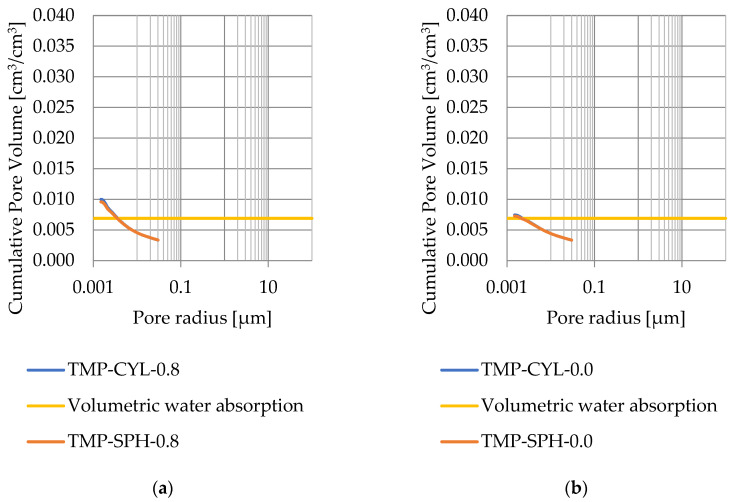
Cumulative pore volume—sample CA1.2 (**a**) 0.8 nm thickness for the nonfreezable layer, (**b**) 0.0 nm thickness for the nonfreezable layer.

**Figure 12:**
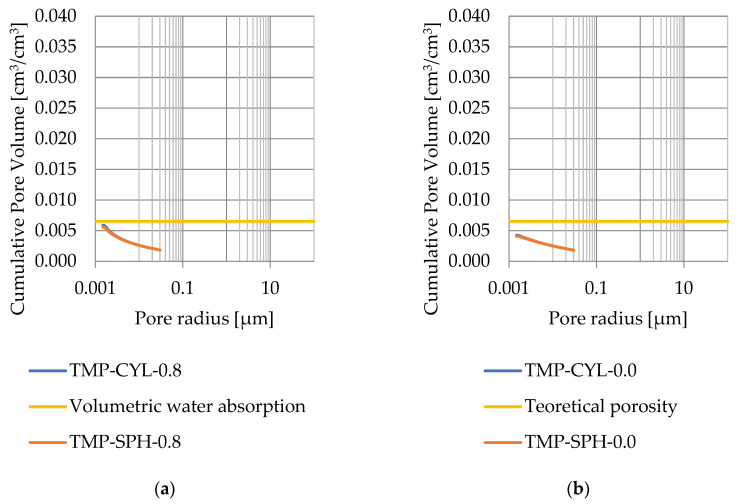
Cumulative pore volume—sample CA2.1: (**a**) 0.8 nm thickness for the nonfreezable layer, (**b**) 0.0 nm thickness for the nonfreezable layer.

**Figure 13:**
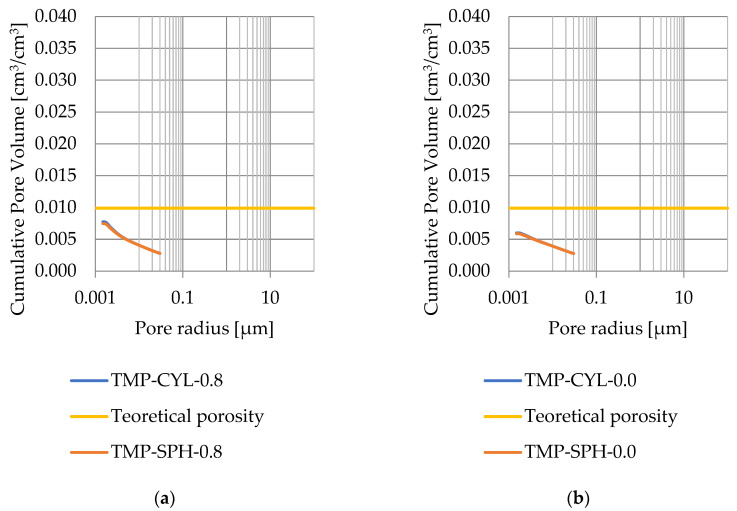
Cumulative pore volume—sample CA2.2: (**a**) 0.8 nm thickness for the nonfreezable layer, (**b**) 0.0 nm thickness for the nonfreezable layer.

Pore size distribution was determined for the tested samples using the thermoporometry method (TMP). The cumulative pore volume calculations were made assuming a spherical shape of the pores and applying Equation (9) for the heat of phase change (as described in Figure 8, Figure 9, Figure 10, Figure 11, Figure 12 and Figure 13, labeled TMP-SPH) and a cylindrical shape of the pores (as described in Figure 8, Figure 9, Figure 10, Figure 11, Figure 12 and Figure 13, labeled TMP-CYL) and assuming Equation (8). Two variants of the thickness of non-freezing water were adopted, equal to 0.8 nm and 0 nm. Additionally, the volumetric water absorption (Section 2.2.2) has been plotted on Figure 8, Figure 9, Figure 10, Figure 11, Figure 12 and Figure 13, which are given in Table 6.

The maximum values of cumulative pore volume calculated for samples DO1 and CA1 using the thermoporometric method give results higher than the volumetric water absorption. In case of the CA1 rock, this effect can be attributed to density of ice taken for pore volume calculations. In case of the DO1, a significant amount of endothermic effects were observed in temperatures under −20 °C, (Figure 8a and Figure 9a), which correspond to a significant increase in cumulative pore volume for pores with a radius under 2 nm. Such results confirm the unusual properties of water in pores with a radius under 2 nm.

**Figure 14:**
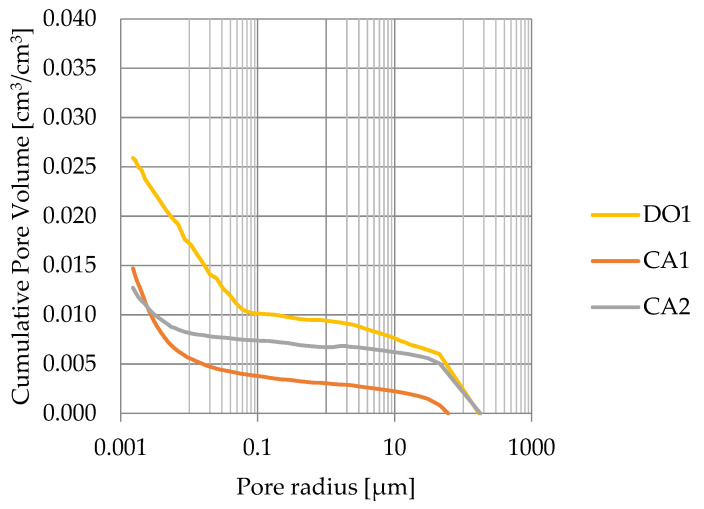
Cumulative pore volume based on mercury intrusion porosimetry.

Porosity obtained from MIP measurements are greater than water volumetric absorption. This indicates that rocks with porosity lesser than 3% have a significant share of closed pores (see Figure 14).

## 4. Conclusions

The research focuses on studying the phase change of water in the pore space of Devonian carbonate rock samples using Differential Scanning Calorimetry (DSC) over a temperature range from −80 to 0 °C. For this purpose, one dolomite and two limestone samples with a porosity of less than 3% were used. Conclusions of this investigation include:

(1) For all rocks examined using the DSC method, a significant share of endothermic effects was observed in the range of −30 to −2 °C. The higher the phase change energy in this temperature range, the greater the observed share of ice calculated on the basis of endothermic effects to the amount of water. For the dolomite DO1, regardless of the adopted equation for the heat of phase change, the calculated amount of ice is greater than the amount of water. This fact suggests that the values of the heat of phase change in some systems may be higher than 334.2 J/g.
(2) The thickness of the non-freezing water layer has the greatest impact on the cumulative pore volume calculated using the TMP method. The smallest difference between the theoretical porosity and the accumulated pore volume obtained by the TMP method is obtained by assuming a constant value of the heat of phase change and zero thickness of the non-freezing layer. The calculated pore volume corresponding to the melting of ice is particularly sensitive to assumptions about the thickness of the non-freezing layer of adsorbed water.
(3) The obtained conclusions suggest the need for further research on the phase change of water in a porous medium and the thickness of non-freezable water.

## Figures and Tables

**Table 1:** Characteristics of the rock samples.

Rock Code	Material Description
DO1	Fine-crystalline ankerite-calcite dolomite rock with a crystalline structure and massive texture (Figure A1). The dolomite rock is composed of ankerite (CaFe(CO_3_)_2_) with an admixture of dolomite. The rock’s volume consists of 80% crystals 30 to 90 μm in size.
CA1	Microsparitic limestone with calcite veins, characterized by a very fine crystalline structure and a random, massive texture (Figure A1). The sample is 100% composed of calcite (Figure A2); 55% of it is microspar (crystals with a diameter of 5–10 μm).
CA2	The calcite content in the rock is over 95% (Figure A2). Quartz is also detected, in small amounts, and calcite veins are observed in optical microscopy. Minerals sized between 15–60 μm constitute 97% of the sample.

**Table 2:** Physical properties of rocks.

Rock Code	Density— MIP (kg/m^3^)	Density— Hydrostatic Method (kg/m^3^)	Density— Le Chatelier * (kg/m^3^)	Apparent Density—MIP (kg/m^3^)	Apparent Density—Hydrostatic Method (kg/m^3^)	Porosity—MIP (%)	Porosity—Hydrostatic Method (%)
DO1	2839	2840	2827	2765	2772	2.59	2.41
CA1	2708	2718	2717	2675	2679	1.24	1.43
CA2	2724	2724	2710	2688	2681	1.49	1.57

* mean value from 3 samples.

**Table 3:** Mass of rocks for DSC measurements.

Rock Code	Sample Code	*m_s_*—Dry Mass (g)	*m_w_*—Water Mass (g)
DO1	DO1.1	22.4863	0.1669
	DO1.2	24.0267	0.1673
CA1	CA1.1	27.4902	0.0540
	CA1.2	26.8650	0.0686
CA2	CA2.1	27.6880	0.0668
	CA2.2	27.4585	0.1008
	*m_s_*—dry mass of the rock, *m_w_*—mass of water in the sample, calculated on the basis of the difference between the mass of the sample saturated with water and the mass of *m_s_*.		

**Table 4:** Phase change energy of melting.

Sample Code	Total energy (J/g)	Energy for *T* < −20 °C (J/g)	Energy for −20 °C ≤ *T* < −2 °C (J/g)	Energy for *T* > −2 °C (J/g)
DO1.1	2.578	0.313	0.376	1.889
DO1.2	2.526	0.133	0.461	1.922
CA1.1	0.656	0.026	0.249	0.380
CA1.2	0.828	0.053	0.331	0.443
CA2.1	0.466	0.039	0.179	0.247
CA2.2	0.668	0.033	0.250	0.384

**Table 5:** Total mass of ice.

Sample Code	The Mass of Water in the Sample (g)	Calculated Total Ice Mass (g)		The Ratio of the Calculated Total Ice Mass to the Water Mass *m_ice-total_*/*m_w_* (g/g)	
		A Variant	B Variant	A Variant	B Variant
DO1.1	0.1669	0.1744	0.1770	1.06	1.06
DO1.2	0.1673	0.1820	0.1844	1.09	1.10
CA1.1	0.0540	0.0542	0.0552	1.00	1.02
CA1.2	0.0686	0.0669	0.0686	0.98	1.00
CA2.1	0.0668	0.0388	0.0400	0.58	0.60
CA2.2	0.1008	0.0551	0.0563	0.55	0.59

**Table 6:** Theoretical porosity for rocks samples for DSC measurements.

Rock Code	Sample Volume (cm^3^)	Volume Occupied by Water (cm^3^)	Volumetric Water Absorption (%)
DO1.1	8.13	0.167	2.05
DO1.2	8.75	0.167	1.91
CA1.1	10.28	0.054	0.53
CA1.2	10.04	0.069	0.68
CA2.1	10.30	0.067	0.65
CA2.2	10.22	0.101	0.99

**Figure A1:**
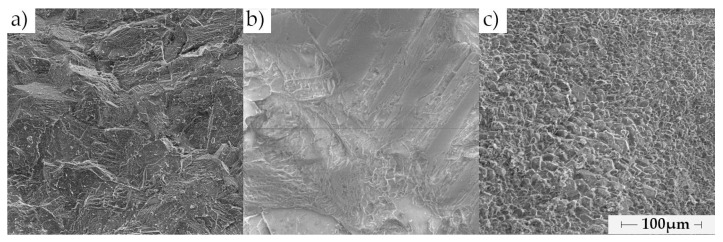
SEM micrographs (800× magnification) (**a**) rock DO1, (**b**) rock CA1, (**c**) rock CA2.

**Figure A2:**
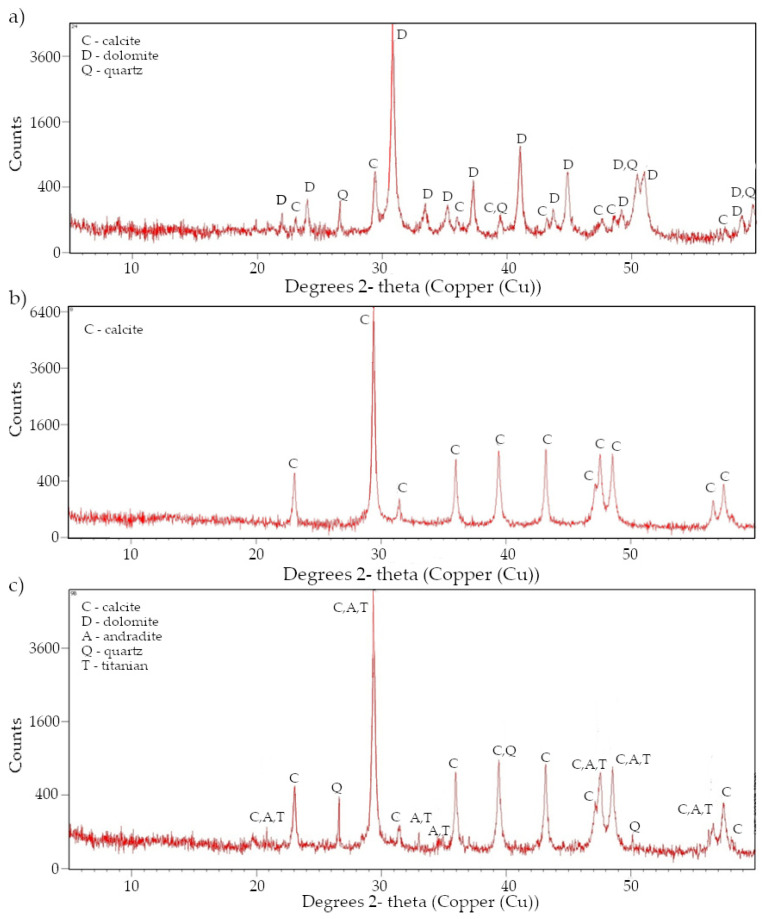
X-ray diffraction pattern (**a**) rock DO1, (**b**) rock CA1, (**c**) rock CA2.

## Data Availability

Data are contained within the article.

## Abbreviations

DSC	differential scanning calorimetry method
*E_SUM_*	total energy of phase change
*E_i_*	amount of energy of phase change at temperature *T_i_*
*L*(*T*)	heat of phase change
MIP	mercury intrusion porosimetry method
*m_ice-total_*	cumulative calculated cumulative
*m_s_*	dry mass of the rock
*m_w_*	water mass
*n_v_*	volumetric absorption
*P*	porosity
*r_cl_*	radius of liquid–solid interphase curvature
*r_gl_*	radius of gas–liquid interphase curvature
*r_p_*	pore radius
*r*_1_, *r*_2_	radii of interface curvature.
*T*	temperature
*T*_0_	freezing temperature of bulk liquid 273.2 K
TMP	thermoporometry method
*V_freezable_*	volume occupied by freezing water,
*V_l_*	volume of liquid
*V_nonfreezable_*	volume occupied by non-freezing water strongly adsorbed to the pore surface
*V_s_*	volume of skeleton
*V_sample_*	sample volume
*γ* *_lc_*	solid–liquid surface tension
*γ_cl_*	surface tension at the solid–liquid interface,
*γ_cg_*	surface tension at the solid–gas interface,
ΔT	difference between the temperature *T* and the temperature 273.2 K
*θ*	contact angle
*ν_m_*	liquid density
*ρ_s_*	density
*ρ_w_*	water density
*ρ_l_*	density of liquid
